# A RULA-Based Comparison of the Ergonomic Risk of Typical Working Procedures for Dentists and Dental Assistants of General Dentistry, Endodontology, Oral and Maxillofacial Surgery, and Orthodontics

**DOI:** 10.3390/s22030805

**Published:** 2022-01-21

**Authors:** Fabian Holzgreve, Laura Fraeulin, Werner Betz, Christina Erbe, Eileen M. Wanke, Dörthe Brüggmann, Albert Nienhaus, David A. Groneberg, Christian Maurer-Grubinger, Daniela Ohlendorf

**Affiliations:** 1 Social Medicine and Environmental Medicine, Institute of Occupational Medicine, Goethe-University, 60590 Frankfurt, Germany; fraeulin@med.uni-frankfurt.de (L.F.); wanke@med.uni-frankfurt.de (E.M.W.); brueggmann@med.uni-frankfurt.de (D.B.); arbsozmed@uni-frankfurt.de (D.A.G.); maurer-grubinger@med.uni-frankfurt.de (C.M.-G.); ohlendorf@med.uni-frankfurt.de (D.O.); 2 Department of Dental Radiology, Institute of Dentistry, Goethe-University, 60590 Frankfurt, Germany; w.betz@em.uni-frankfurt.de; 3 Medical Center of the Johannes Gutenberg, Department of Orthodontics, University Mainz, 55131 Mainz, Germany; erbe@uni-mainz.de; 4 Principles of Prevention and Rehabilitation Department (GPR), Institute for Statutory Accident Insurance and Prevention in the Health and Welfare Services (BGW), 20095 Hamburg, Germany; albert.nienhaus@bgw-online.de

**Keywords:** ergonomics, kinematic analysis, musculoskeletal disorders, risk assessment, inertial motion capture, inertial sensors

## Abstract

Background: In general, the prevalence of work-related musculoskeletal disorders (WMSD) in dentistry is high, and dental assistants (DA) are even more affected than dentists (D). Furthermore, differentiations between the fields of dental specialization (e.g., general dentistry, endodontology, oral and maxillofacial surgery, or orthodontics) are rare. Therefore, this study aims to investigate the ergonomic risk of the aforementioned four fields of dental specialization for D and DA on the one hand, and to compare the ergonomic risk of D and DA within each individual field of dental specialization. Methods: In total, 60 dentists (33 male/27 female) and 60 dental assistants (11 male/49 female) volunteered in this study. The sample was composed of 15 dentists and 15 dental assistants from each of the dental field, in order to represent the fields of dental specialization. In a laboratory setting, all tasks were recorded using an inertial motion capture system. The kinematic data were applied to an automated version of the Rapid Upper Limb Assessment (RULA). Results: The results revealed significantly reduced ergonomic risks in endodontology and orthodontics compared to oral and maxillofacial surgery and general dentistry in DAs, while orthodontics showed a significantly reduced ergonomic risk compared to general dentistry in Ds. Further differences between the fields of dental specialization were found in the right wrist, right lower arm, and left lower arm in DAs and in the neck, right wrist, right lower arm, and left wrist in Ds. The differences between Ds and DAs within a specialist discipline were rather small. Discussion: Independent of whether one works as a D or DA, the percentage of time spent working in higher risk scores is reduced in endodontologists, and especially in orthodontics, compared to general dentists or oral and maxillofacial surgeons. In order to counteract the development of WMSD, early intervention should be made. Consequently, ergonomic training or strength training is recommended.

## 1. Background

Dental professionals frequently suffer from work-related musculoskeletal disorders (WMSD). Both dentists and dental assistants have a high prevalence of WMSD, especially in the neck and the trunk as well as in the upper limbs [1,2,3,4,5]. It has been suggested that forced postures that are statically maintained during the dental work are a major hazard for WMSD in dental professionals [1,2,4,6]. While the patient is lying supine on the treatment chair, the dentist (D) and dental assistant (DA) sit adjacent to the patient, and are required to gain a sufficient line of sight into the narrow mouth. A static neck and trunk flexion of more than 30° [7,8,9,10], a simultaneous lateral flexion and/or rotation to the right in the trunk [9,10,11], and shoulder abduction [7] have been described as typical working postures in dental professionals.

However, in the available kinematic investigations, scarcely any differentiation between the specialized treatment procedures has been explored [12]. As the dental treatment profile is vast, specializations are common; for example, generalists, orthodontists, oral surgeons, and endodontologists apply different techniques, and their typical working days are scarcely comparable. It can be expected that the dental task (e.g., extraction of a tooth or the preparation of a tooth) determines the posture, and the duration that the posture is maintained, within that task. However, it can be shown that the performance of the same activities within four different dental work concepts does not cause any noteworthy posture-relevant differences [13].

Although there are numerous studies on the prevalence of WMSD and ergonomic interventions [1,2,3,5,9,12,14,15,16,17,18,19,20,21,22,23,24,25,26,27], differentiations between the fields of specialization (e.g., general dentistry, endodontology, oral and maxillofacial surgery, or orthodontics) in these studies are rare [28,29]. Uppada et al. [29], for example, found that oral surgeons show a higher prevalence of WMSD than generalists, while Newell and Kumar [28] argued for a different ergonomic load in orthodontists compared to generalists. According to both research, generalists work on posterior molars and, therefore, have to spend more time in a bent-over position while orthodontists work rather superficially and can maintain a more upright posture [28,30]. Meanwhile, it remains unclear whether orthodontists suffer less from WMSD than generalists [30], or if the extent is similar [20].

For all fields of specialization, one may speculate that the patient’s head is arranged in such a way that the dentist has the best possible sight into the mouth. In four-handed dentistry, however, the DA for the majority of their time sits opposite the D and, potentially, has to sacrifice posture to a greater extent than the D in order to see into the mouth. Although, to our knowledge, this has not been compared before, survey data show that DAs have a higher prevalence of WMSD than Ds [24,31], and that different body regions are affected [9].

In general, the prevalence of WMSD in dentistry is high [1,3,4,32,33] and WMSD are a major cause for ill-health retirement [16,34]. The characteristics of dental work are known to be responsible for an increased prevalence of WMSD in dentistry, such as the profession of dentistry per se, missing breaks, the work schedule, the number of patients treated and certain activities, such as teeth polishing [1]. Thus, it is crucial to gain more insight as to whether there are differences in the ergonomic risks of the different fields of specialization.

The Rapid Upper Limb Assessment (RULA) [35] is a well-known method for the evaluation of the ergonomic risk as, in addition to assessing the neck, trunk, and upper limb postures, it also considers whether a working process has static or dynamic movement sequences. Lately, an application of the original paper–pen method for an automated use of kinematic data has been published by Vignais et al. [36,37]. This offers an objective and precise approach for the evaluation of the ergonomic risk in real-world conditions with inertial motion capture (IMC) [38]. The rapid development of IMC systems has rendered the captured data sufficiently reliable and precise for scientific application [39,40].

This study is part of the SOPEZ project: “Study for the optimization of ergonomics in dental practice” [6], having the current aim to compare the ergonomic risk of Ds and DAs in the practices of oral surgeons, endodontologists, general dentists, and orthodontists when performing typical dental procedures. Parts of this project with dental students [41] have already been published. 

The character of the current investigation is clearly explorative, nevertheless, two main research questions were to be answered: (a) Is there a difference in the ergonomic risk between the four dental fields of specialization for Ds and DAs, respectively? and (b) Is there a difference regarding the individual field of specialization in the ergonomic risk between the Ds and DAs? 

## 2. Materials and Methods

### 2.1. Subjects

In total, 60 dentists (33 male/27 female) and 60 dental assistants (11 male/49 female) were recruited to take part in this study. In order to represent the fields of dental specialization, the sample was composed of 15 dentists and 15 dental assistants from each of the fields of endodontology, oral and maxillofacial surgery, general dentistry, and orthodontics. The sociodemographic data can be seen in Table 1. The only eligibility for participation was the requirement for subjects to be right-handed. However, exclusion criteria comprised severely restrictive malformations of the spine, injuries to the musculoskeletal system (e.g., slipped vertebra or urgent herniated discs), stiffened spinal joints, rheumatic diseases, and relevant surgery in the previous two years.

### 2.2. Dental Tasks

In order to reflect realistically the typical dental tasks representative for each field of specialization, standardized working procedures were conducted. In each treatment procedure, the most common tasks were included that the dentists would normally execute multiple times during a typical working day. In order to thoroughly assess stresses to the musculoskeletal system within dental tasks, each of the dental quadrants had to be represented in the standardized procedures (Table 2). Magnification loupes were used for endodontology and general dentistry as it is common to work with this equipment in these fields. 

### 2.3. Measurement System

**Figure 1:**
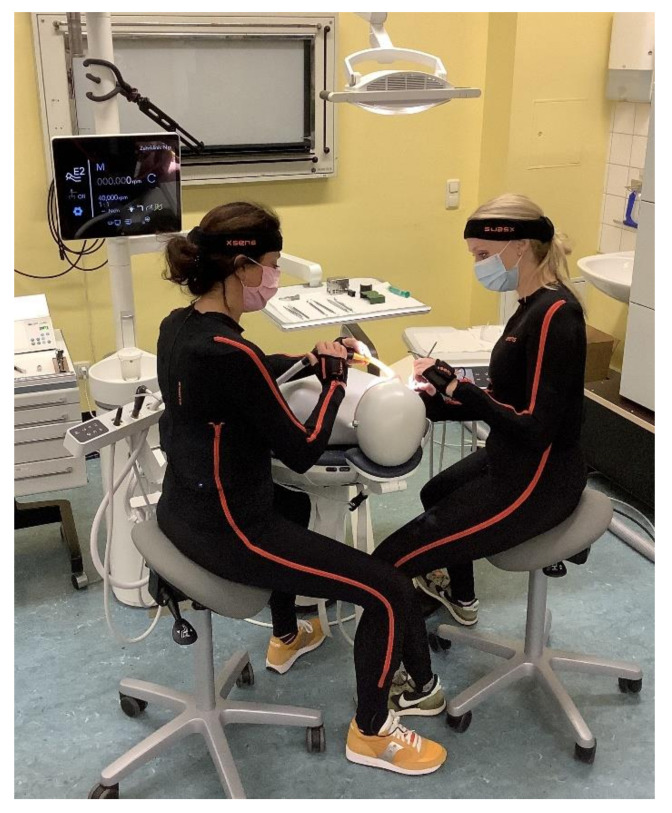
Joint treatment of dentist (right position) and dental assistant (left position) on the dummy head. Both wear the Xsens lycra-suit with seventeen integrated sensors.

The inertial motion capture system MVN Link by Xsens (Enschede, Netherlands) was used in all kinematic recordings (Figure 1). The system provides a sampling rate of 240 Hz; however, for this investigation, 24 Hz were considered to be suitable since there were scarcely any high-speed movements. The personal inertial measurement system enables recordings in the field in real-working scenarios and can be used in comparison to optical measurement systems without complex set-up. Further details have been published in Ohlendorf et al. [6]. All recordings were obtained using the ‘No Level’ scenario, a mode in which the limbs and segments are considered relative to the pelvis. The ‘No Level’ scenario is provided in the Xsens Analyze software and offers the best data quality for ergonomic analyses.

### 2.4. Rapid Upper Limb Assessment (RULA)

**Figure 2:**
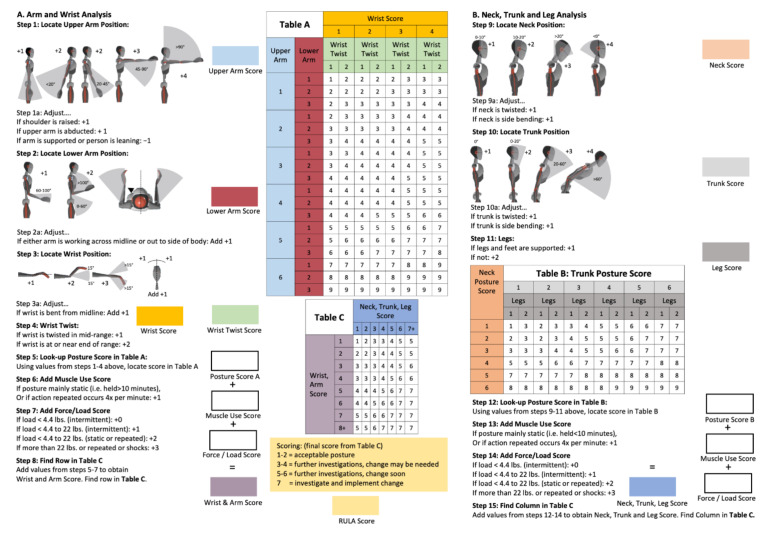
The Rapid Upper Limb Assessment (RULA) Worksheet [38]. The “(**A**) Arm and Wrist Analysis” consists of 8 steps (steps 1–8), which are added to the “Wrist and Arm Score” (left side). On the right side “(**B**) Neck, Trunk and Leg Analysis”, steps 9–15 are added to the “Neck, Trunk, and Leg Score”. The combination of the “Wrist and Arm Score” and the “Neck, Trunk, and Leg Score” using table C leads to the final RULA Score.

As with other observational methods for a quick ergonomic risk assessment application in occupational health, the original version of the RULA [35] was created as a form to be filled-in by the participant. By applying this scoring method, experienced occupational health staff can rapidly decide whether their working procedures are ergonomically risky. The worksheet consists of 15 steps (Figure 2) in which the position of the limbs, neck, and the trunk are assessed and evaluated according to the risk potential. In the RULA, the static or dynamic natures of postures are considered; this is especially interesting for dentistry since the static postures are considered a major health hazard.

In total, the steps add up to an overall score summarizing the ergonomic risk: score 1–2: acceptable risk; score 3–4: further investigation, change may be needed; score 5–6: further investigation, change soon; score 7: investigate and implement change.

### 2.5. Measurement Protocol

When the subjects arrived at the recording site, they were equipped with the measurement apparatus and the calibration procedures performed (neutral pose + walking sequence). The dental treatment procedures were performed in a laboratory setting at the Institute for Occupational Medicine at the Goethe University Frankfurt since the inventory at dental practices (the treatment chair, trays, and carts) are not always arranged in a standardized manner [43,44,45]. In addition, the working environment was created anew for each participant. The treatment pairs of the D and DA performed their protocol using a dummy head. All participants were allowed to work at their own preferred speed. 

### 2.6. Data Analysis

The recorded data were first processed in the MVN Analyze software, provided by Xsens Technologies. Only reliable data samples were extracted and compiled further into mat.-files. The RULA coding system was in some parts slightly modified by transcripting into a Matlab code (Mathworks, Version 2020a). This modification was necessary since not all steps were suitably designed for the application to objective kinematic data; details are published in Maurer-Grubinger et al. [38]. In the current study, we chose to evaluate the data in a drop-down manner, using several levels of complexity. For the most global approach, we used the median of the final RULA score. Since we recorded entire work processes lasting over several min, we were further able to determine how much time the subjects spent relatively in each RULA score (scores 1–7), thus obtaining the *relative average risk score over the time (Rel. av. RST)*. This enables a more precise view of the ergonomic hazard to distinct body parts for the right- and left-hand body sides, respectively. At this level we included step 1 (*upper arm score*), step 2 (*lower arm score*), and a combination of steps 3 and 4, which was suggested by Vignais et al. [36] (*wrist score*), as well as step 9 (*neck score*) and step 10 (*trunk score*). Since the maximum achievable scores vary for the different steps, we also calculated the *ergonomic risk potential* (ERP); this meant that we could calculate how much working time was spent relatively in the maximum achievable score and, thus, obtain a better comparability.

An evaluation based on the combination of RULA and inertial motion capture [38] data allows the evaluation of three outcome variables:

median + interquartile distance (IQD)
relative average risk score over the time (Rel. av. RST)
ergonomic risk potential (ERP)

We calculated the relative average risk score over the time as follows:

Relative time spent at RULA score 1 × 1 + relative time spent at RULA score 2 × 2 + relative time spent at RULA score 3 × 3(..) + relative time spent at RULA score 7 × 7.

The RULA steps and body regions (local scores) are listed as follows:

1. Neck Score            - RULA Step 9
2. Trunk Score             - RULA Step 10
3. Upper Arm Score (left and right)   - RULA Step 1
4. Lower Arm Score (left and right)   - RULA Step 2
5. Wrist Score (left and right)     - RULA Steps 3 + 4

## 3. Statistical Analysis

Socio-demographic data were calculated for each field of specialization, respectively. For this purpose, data were tested for normal distribution using the Kolmogorov–Smirnov–Lilliefors test. Since the subject’s data were normally distributed, the mean and standard deviation were calculated. The kinematic data were not normally distributed. Therefore, non-parametric tests were applied. For calculating the differences between the fields of dental specialization, the Kruskal–Wallis Test with multiple comparisons was employed. 

Due to the exploratory approach in evaluating the kinematic data, a Bonferroni correction was not applied.

The significance level was set at α = 5%.

## 4. Results

### 4.1. Differences between the Fields of Dental Specialization

**Figure 3:**
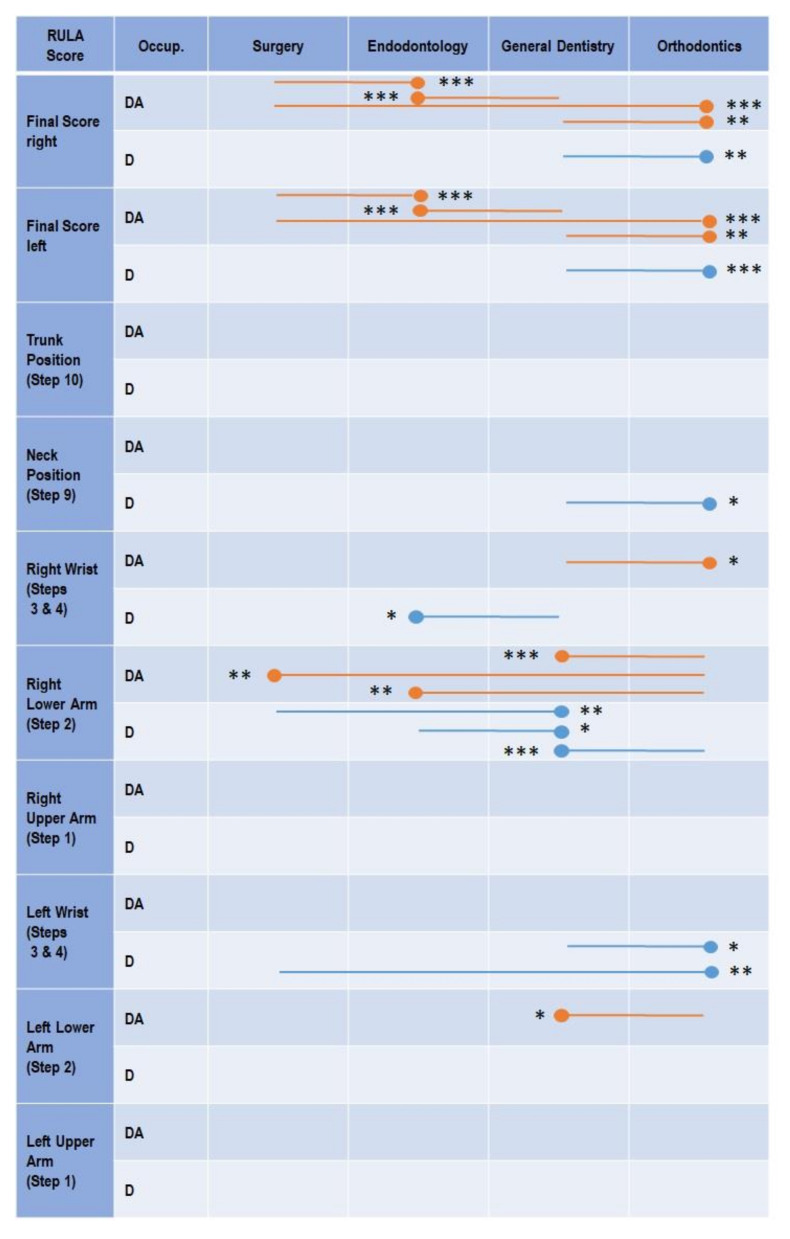
Significant differences in the *relative average risk score over the time* between the four specializations for DAs (orange lines) and Ds (blue lines) in the outcomes of *final score* right and left; *trunk score*, *neck score*, *wrist score* right and left; *lower arm score* right and left; and *upper arm score* right and left. The lines indicate the comparisons calculated (e.g., general dentistry—orthodontics). The circle marks the field of specialization with the significantly lower (better) ergonomic risk score, while the asterisks indicate the statistical significance (* *p* < 0.05; ** *p* < 0.01; *** *p* < 0.001).

**Figure 4:**
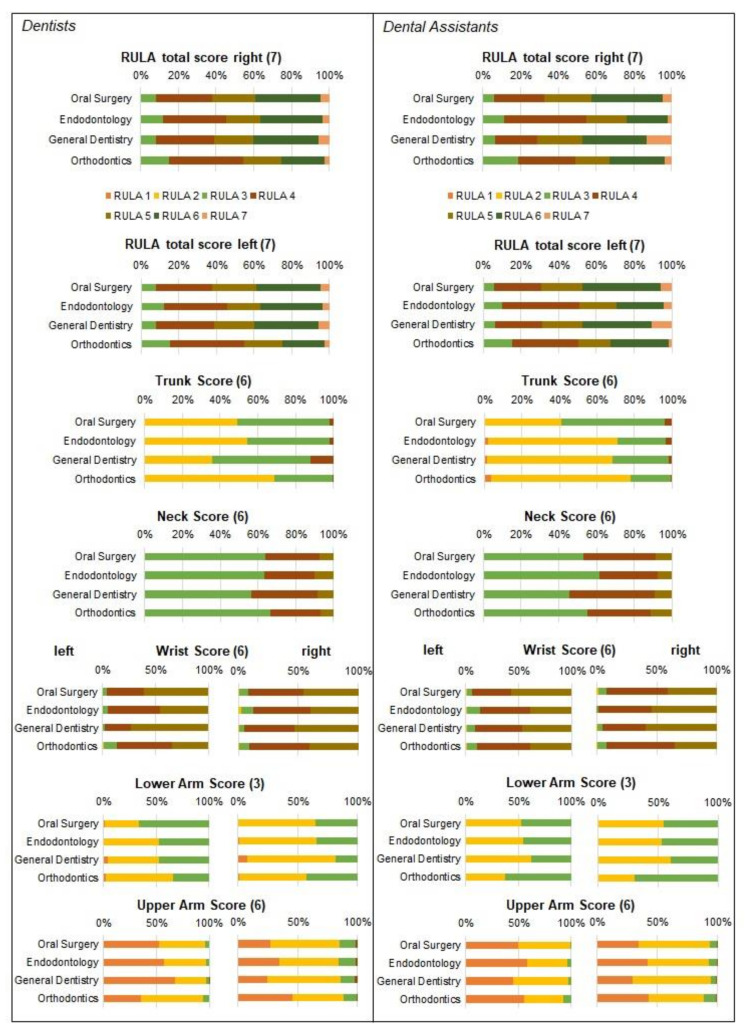
The figure illustrates the relative shares of the different RULA scores for dentists (**left**) and dental assistants (**right**). All subfigures show for each body region the relative shares of the different RULA scores for all four dental fields of dental specialization and use the same color legend, shown under “RULA total score right”. The maximum possible RULA score of each region is specified in brackets.

The results of the ergonomic analysis are displayed in Table 3 and the differences between the fields of specialization are shown in Figure 3. The distribution of the *relative average risk score over the time* is displayed in Figure 4.

### 4.2. Dental Assistants

The *final score* ERP for the DAs in all fields of specialization ranged for the right body side between 66.54 and 74.7%, and for the left body side between 66.11 and 75.15% (Table 3). Main differences were found between the different fields of specialization for the *final score*. Here, dental tasks in endodontology and orthodontics posed, for the right and left body side, a highly significant, lower risk to the DAs than the tasks of oral and maxillofacial surgery or general dentistry (Figure 3). These differences are also reflected in the respective RULA value shares of the studied occupational groups. While the DAs in endodontology and orthodontics had greater shares of RULA 3 and 4 (both about 55%), in general dentistry and oral and maxillofacial surgery, the DAs worked for almost 75% of the time in RULA scores 5, 6, and 7 (Figure 4) in both body sides, respectively. In summary, the total ergonomic risk does not differ between the body sides, but shows a significantly greater exposure to high risks in oral surgery and general dentistry, compared to endodontology and orthodontics.

The kinematic RULA scores revealed that for all fields of specialization, the highest ergonomic risks occurred in both lower arms (79.1–89.7% ERP), followed by the wrists (69.5–76.1% ERP), the neck (57.6–61.4% ERP), and the trunk (36.2–44.3% ERP), whilst the dental tasks posed the least risk for the upper arms (24.9–29.9% ERP).

Comparing the dental fields of dental specialization, only a few significant differences occurred in the lower arms and wrists. Compared to the DAs in general dentistry, the DAs in orthodontics showed a slightly lower ergonomic risk for the right wrist (orthodontics: 69.5% ERP; general dentistry: 76.0% ERP), but also a slightly higher risk in the lower left arm (orthodontics: 87.1% ERP; general dentistry: 79.3% ERP). In the lower right arm, however, the DAs in orthodontics showed a higher ergonomic risk than all other fields of specialization (orthodontics: 89.7% ERP; oral and maxillofacial surgery: 81.7% ERP; endodontology: 81.7% ERP; general dentistry: 79.1% ERP).

### 4.3. Dentists

The magnitude of the ERP of the *final score* in dentists was comparable to the DAs. The analysis revealed in all fields of specialization, ERP values between 66.57 and 72.13% (right), and 64.08 and 74.1% (left), of the maximum achievable *final score* (Table 3). However, orthodontists showed slightly, but significantly, lower ergonomic risk than general dentists in both body sides (Figure 3). Analogue to DAs, these differences are also reflected in the respective RULA value shares of the studied occupational groups. While orthodontics spent about 25% of the time in high RULA scores (6 and 7), oral and maxillofacial surgeons, general dentists spent almost 40% in a high ergonomic risk (Figure 4).

Regarding the kinematic RULA score, all fields of specialization showed similar risks in the body segments. The regions with the highest ergonomic risk were the right and left lower arm (70.8–88.8% ERP) followed by the wrists (69.5–78.5% ERP), the neck (56.2–58.7% ERP), the trunk (38.7–45.5% ERP), and the upper arms (22.6–31.8% ERP). However, slight statistically significant differences were found as follows: orthodontists showed an advantage in the neck area (56.2% ERP) and in the left wrist (69.5% ERP) compared to general dentists (neck: 58.7% ERP; left wrist: 78.5% ERP). The left wrist of orthodontists was also at a lower risk compared to oral and maxillofacial surgeons (75.5% ERP). In the lower right arm, general dentists (70.8% ERP) showed the least ergonomic risk compared to all other three fields of specialization (endodontists: 76.9%; oral and maxillofacial surgeons: 78.1% ERP; orthodontists: 80.4% ERP). Concerning the right wrist, endodontists (70.0% ERP) were slightly superior to the general dentists (75.2% ERP).

### 4.4. Differences in the Ergonomic Risk between Dentists and Dental Assistants According to Their Field of Dental Specialization

The results of the Mann–Whitney-U test are displayed in Table 3. This shows that the ergonomic risk for both occupations are, mostly, very similar. However, in some cases, mostly in the arms, slight but significant differences were observed and, in some cases, differences were only significant in the median scores; here, we present only those results that were significant in the *relative average risk score over the time*. The percentage of time in which the maximum possible score (ERP) was reached are displayed (in brackets) along with the *p*-values.

### 4.5. Oral and Maxillofacial Surgery

Here, the Ds worked significantly with a lower ergonomic risk than the DAs both in the right and left body side: *final score* right: DAs: 74.12% ERP; Ds: 71.49% ERP; *p* = 0.045; *final score* left: DAs: 74.59% ERP; Ds: 71.13% ERP; *p* = 0.013. However, in the upper right arm, the DAs showed a slightly lower ergonomic risk than the Ds (DAs: 29.0% ERP; Ds: 31.6% ERP; *p* = 0.033).

### 4.6. Endodontology

In the right wrist, the Ds reached the maximum possible score with a slightly lower percentage than the DAs (DAs: 75.31% ERP; Ds: 70.0% ERP; *p* = 0.033). However, in the upper right arm, the DAs were slightly superior to their colleagues (DAs: 27.8% ERP; Ds: 31.8% ERP; *p* = 0.01). 

### 4.7. General Dentistry

Here, in the lower right arm, the Ds reached the maximum possible RULA score at a lower percentage than the DAs (DAs: 79.1% ERP; Ds: 70.8% ERP; *p* = 0.001).

### 4.8. Orthodontics

Here, the Ds showed a lower ergonomic risk in the neck (DAs: 60.4% ERP; Ds: 56.2% ERP; *p* = 0.002), the lower right arm (DAs: 89.7% ERP; Ds: 80.4% ERP; *p* < 0.001), and the lower left arm (DAs: 87.1% ERP; Ds: 76.0% ERP; *p* < 0.001) than the DAs.

## 5. Discussion

The objectives of the present analysis were to examine the differences in ergonomic risk in four dental specializations for Ds and DAs, and to determine whether there is a difference in the ergonomic risk between Ds and DAs in each specialization.

Regarding the *final score* of both the right and the left body side, the DAs especially showed higher differences within the fields of specialization. The results revealed significantly reduced ergonomic risks in endodontology and orthodontics compared to oral and maxillofacial surgeons and general dentistry (Figure 3). Dentists showed comparable results regarding the *final score* with a reduced magnitude of difference, revealing significant differences only between orthodontics and general dentistry (Figure 3). Nevertheless, also for dentists, the values of the ERP indicate a clear trend that the ergonomic risk in endodontology and orthodontics is substantially lower than in oral and maxillofacial surgeons and general dentistry (Table 3).

With regard to orthodontists, it must be taken into account that the results can diverge in cases involving the insertion of lingual or palatally inserted appliances or, for example, TPA, Quadhelix, or GNE; nevertheless, this comparison does not form part of the present study.

However, these results were not observed in the individual body segments. Concerning the dentists, specialization differences are present in the neck, right wrist, upper right arm, and left wrist, with orthodontists having the lowest scores (Figure 3). However, for the dental assistants, differences in the right wrist and right and left forearm tended to be reversed; almost universally, the score here was worse for orthodontic assistants than for other specialties (except of the right wrist). For the trunk and the right and left forearm, there are no differences between the four specializations in either profession. In this context, it must be taken into account that right-handed persons were analyzed and, thus, the right hand is also the leading working hand.

If all significant differences between the specializations of the Ds and DAs are considered together, the *relative average risk score over the time* for the *final score* right and left and the individual segments in the field of endodontics and orthodontics is almost always lower than in the other specializations. In oral and maxillofacial surgery, on the other hand, the *relative average risk score over the time* is almost always the highest. The reason for this may be the fact that both specialties (oral and maxillofacial surgery and general dentistry) involve more precision work than the other specialties.

Whether this ergonomic risk assessment of the individual specializations corresponds to the prevalence of WMSD cannot yet be concluded. In the literature, it is controversial whether the RULA values are directly linked to WMSD [37,46,47]. With regard to the prevalence of WMSD, most surveys show a differentiation between generalists and orthodontists; according to these, the WMSD prevalence is lower among orthodontists [12]. Although differentiated data have also been collected in a survey between Ds and DAs, details about the specialization direction were not probed [12]. The lifetime, 12-month and 7-day prevalence were greater among the DAs than among the Ds; this has been found to be true for the whole body as well as for the individual body regions [1,4,5]. Differentiated data per discipline would be interesting to explore, since in the specialty of orthodontics, followed by endodontology, the least work is performed (in percentage terms) at the highest ergonomic risk for the neck and trunk area. With regard to the orthodontic working method, these results can be explained by the fact that in the selected activities, the appliances are attached vestibularly, and so there is less need to lean forward and rotate than when working more palatally. This assumption should be explored more deeply in further analyses. WMSD prevalence in the previous 12 months in the neck and trunk region (85–48%) for DAs and (71–33%) for Ds [1,4,5] are also fundamentally higher in dentistry, per se, than in the German general population [48]. The prevalence for WMSD in Ds and DAs is also high for the wrists [49]; these are ergonomically very stressed in all specializations, as evidenced by the high RULA scores (Figure 4; Table 3) due to the incessant fine motor movements that predominate here. However, comparative data for the general population are not available.

In the case of the activities analyzed in this study, it must also be borne in mind that these were selected from the spectrum of the respective specializations after consultation with specialist dentists and, therefore, only reflect a proportion of the possible dental treatment options. In addition, these treatments were performed on a dummy head and not on an actual patient, which may have influenced the results [6]. Furthermore, this study assumes that there are defined work patterns for the Ds and DAs in the different specializations; however, this does not necessarily reflect reality, as each team of practitioners has a distinct individual pattern of activities and procedures. Consequently, this study is a laboratory study and not a field analysis. The present study design was chosen so that the same conditions were available for each participant (per specialization) and, thus, comparability could be established. However, it must also be taken into account that the individual specialty-specific dental activities performed on the dummy head were not comparable with each other; this comparison was not the aim of the present analysis. Rather, it should be possible to produce a more detailed statement, on the one hand, as to how high the prevalence for WMSD is in Ds and DAs, and, on the other hand, whether these differ in the various specialist disciplines. The latter should demonstrate the versatility of dental treatment options and specify them by means of a kinematic analysis using an ergonomic risk analysis by RULA. However, the diversity of dental treatment options is less evident in the overall score and in the individual segments; for example, the *relative average risk scores over the time* vary minimally in the left and right upper and lower arms and the left and right wrist.

The comparison of Ds and DAs per specialization reveals isolated significant differences; although, no clear trends can be identified. Moreover, these absolute differences are also so small that they are likely to have little clinical relevance. This more detailed insight into the way of working may possibly be used for career starters with handicaps in order to advise them against a particularly burdensome specialization. However, this advice would then have to be given in consideration of the specific results for certain body regions. Nonetheless, in this way, there is the possibility that later occupational restrictions due to complaints in the musculoskeletal system could be less severe. Despite the fact that in the field of oral and maxillofacial surgery, the scores were sometimes higher than those of other specialties, all dental career starters should carry out systematic muscle building training, targeted at the weak points of the body, in order to counteract the static postures adopted with selected dental tasks.

In order to be able to represent dental activity in the best possible way, an activity protocol was developed. To ensure validity, the subjects were allowed to apply their individual working speed, analogous to daily patient treatment. Since median and relative risk distributions were determined in the evaluation of the kinematic data, the working speed had no influence on the results. One aspect that was not been considered in this study is the use of magnifying glasses. During dental treatment, these could positively favor the working posture since dental work is performed with indirect vision [50]. Since not all activities are performed using indirect vision and not all dentists work with it, the use of such means was not permitted in this study. It can, thus, be assumed that less time is spent in ergonomically stressful postures when working with indirect vision, particularly with regard to the trunk and neck. The torso would not have to be bent forward as far, while the neck and head would not have to be rotated or laterally inclined to such an extent. Working with a dental microscope, especially in oral surgery and endodontics, may also reduce working at such a high ergonomic risk score [51,52,53]. Regarding the ergonomic risk when working with indirect vision or a dental microscope, further analysis would need to be performed in the future.

Furthermore, it is also known that one and the same treatment in different quadrants causes a different posture [54]. Therefore, it would also be interesting to consider this aspect more closely in further analyses with regard to ergonomic risk and, in this context, to analyze the behavioral but also the relationship-related aspects that could positively favor the risk.

In principle, laboratory studies, such as the present investigation, have their limitations; nevertheless, the results of the entire SOPEZ project [4,5,6,13,31,38,41] provide important basic data for further investigations. Despite the measurements being made under laboratory conditions, it was, nonetheless, possible to demonstrate the risk potentials for the development and occurrence of WMSD, the high prevalence of which has already been documented in many surveys [1,4,5,9,55]. Future analyses should investigate the effect of parameters such as sex, age, weight, or height on body postures and ergonomics in dentists and dental assistants.

## 6. Conclusions

Within the framework of this quantitative, ergonomic risk assessment of selected dental tasks in the specialization fields of oral and maxillofacial surgery, endodontology, orthodontics, and general dentistry, it could be shown that the ergonomic risk in all dental disciplines reaches moderate to high ergonomic risk scores—for dentists and dental assistants. Nonetheless, minor differences can be observed, according to which the percentage of time spent working in the higher risk scores is lower for orthodontic and endodontic tasks, than for general dentistry or oral and maxillofacial surgery tasks. This is particularly evident in the neck, trunk, and wrist areas. The differences between dentists and dental assistants within a specialist discipline are only less existent.

## Figures and Tables

**Table 1:** Socio-demographics of dentists (D) and dental assistants (DA) for each field of specialization. The mean values with standard deviations are shown.

Field of Specialization	Occup.	Sex	Work Years	Age (Years)	Height (cm)	Weight (kg)
Generalists	D	8 f/7 m	9.1 ± 12.0	37.1 ± 12.1	164.4 ± 47.1	72.1 ± 11.5
	DA	10 f/5 m	5.5 ± 5.0	28.4 ± 6.0	146.6 ± 64.9	70.7 ± 15.1
Oral and maxillofacial surgeons	D	3 f/12 m	9.5 ± 9.0	36.3 ± 9.6	177.8 ± 8.2	75.5 ± 12.0
	DA	12 f/3 m	5.0 ± 2.4	26.7 ± 2.5	172.9 ± 9.2	68.3 ± 7.8
Endodontologists	D	8 f/7 m	6.6 ± 4.3	32.8 ± 4.1	176.4 ± 9.1	68.2 ± 10.1
	DA	14 f/1 m	5.1 ± 6.0	26.2 ± 7.0	163.1 ± 73.2	69.3 ± 9.2
Orthodontists	D	8 f/7 m	6.1 ± 2.7	32.6 ± 3.4	179.2 ± 10.9	69.5 ± 14.4
	DA	13 f/2 m	6.6 ± 4.7	29.1 ± 4.1	158.6 ± 48.3	68.2 ± 12.1

This study was approved by the ethics research committee of the Goethe-University (356/17) in Frankfurt am Main, Germany. All subjects provided written informed consent.

**Table 2:** Dental tasks of the fields of specialization. More detailed information can be found in Ohlendorf et al. [42].

		Task	Quadrant 1	Quadrant 2	Quadrant 3	Quadrant 4
General Dentistry			Tooth filling of tooth 16	Preparation of tooth 25 for crown uptake	Root canal treatment on tooth 36	Tartar removal in the 4th quadrant
	1	D	Prepare tooth cavity with a cylindrical diamond bur and the use of wedges.	Occlusal reduction using an occlusal reducer.	Performing an entrance cavity and trepanation on tooth 36 using a diamond-coated cylinder.	Removal of supra- and subgingival tartar/calculus using scalers and curettes.
	1	DA	Suction during preparation of the cavity.	Suction during preparation and retracting the cheek with a mirror if requested by the dentist.	Suction during preparation and retracting the cheek with a mirror if requested by the dentist.	Suction during curettage.
	2	D	Create a Tofflemire die using a die clamp.	Chamfer preparation using a torpedo-shaped diamond bur and approximal reducer.	Find the channel entrance using an endo file.	
	2	DA	Activating and blending Ketac for the tooth filling using a Ketac-set and mixing device and passing it to the dentist.	Suction during preparation and retracting the cheek with a mirror if requested by the dentist.	Retracting the cheek with a mirror if requested by the dentist.	
	3	D	Tooth filling with Ketac while using a Ketac-set, a cougar/Heidemann and a ball-shaped plugger, followed by the removal of the Tofflemire clamp.		Manual preparation of the canal using an ISO 20–40 endo file with regular irrigation using an irrigation cannula.	
	3	DA	No task.		Suction during irrigation and retracting the cheek with a mirror if requested by the dentist.	
Endodontology			Root canal treatment of tooth 16	Root canal treatment of tooth 26	Root canal treatment of tooth 36	Root canal treatment of tooth 46
	1	D	Application of the rubber dam.	Application of the rubber dam.	Application of the rubber dam.	Application of the rubber dam.
	1	DA	Helping to apply the rubber dam.	Helping to apply the rubber dam.	Helping to apply the rubber dam.	Helping to applicate the rubber dam.
	2	D	Trepanation of the tooth and access preparation including the enlarging of the root canal entrance.	Trepanation of the tooth and access preparation including the enlarging of the root canal entrance.	Trepanation of the tooth and access preparation including the enlarging of the root canal entrance.	Trepanation of the tooth and access preparation including the enlarging of the root canal entrance
	2	DA	Suction during trepanation and possibly the application of a file ISO 20.	Suction during trepanation and possibly the application of a file ISO 20.	Suction during trepanation and possibly the application of a file ISO 20.	Suction during trepanation and possibly the application of a file ISO 20.
	3	D	Root canal preparation with hand files at a certain working length, irrigation after each file and removal of the rubber dam.	Root canal preparation with hand files at a certain working length, irrigation after each file and removal of the rubber dam.	Root canal preparation with hand files at a certain working length, irrigation after each file and removal of the rubber dam.	Root canal preparation with hand files at a certain working length, irrigation after each file and removal of the rubber dam.
	3	DA	Setting the working lengths of the ISO 35, 40, 45 files to 18 mm each and aspirating the rinsing liquid.	Setting the working lengths of the ISO 35, 40, 45 files to 18 mm each and aspirating the rinsing liquid.	Setting the working lengths of the ISO 35, 40, 45 files to 18 mm each and aspirating the rinsing liquid.	Setting the working lengths of the ISO 35, 40, 45 files to 18 mm each and aspirating the rinsing liquid.
	4	D	Removal of the rubber dam.	Removal of the rubber dam.	Removal of the rubber dam.	Removal of the rubber dam.
	4	DA	Help with the rubber dam removal.	Help with the rubber dam removal.	Help with the rubber dam removal.	Help with the rubber dam removal.
Orthodontics			Multiband Treatment	Multiband Treatment	Multiband Treatment	Multiband Treatment
	1	D	Acid etching.	Acid etching.	Acid etching.	Acid etching.
	1	DA	Assisting: applying etching gel, aspiration.	Assisting: applying etching gel, aspiration.	Assisting: applying etching gel, aspiration.	Assisting: applying etching gel, aspiration.
	2	D	Direct bonding of braces on teeth 1, 3, 4, and 6 and opening of self-ligating braces.	Direct bonding of braces on teeth 1, 3, 4, and 6 and opening of self-ligating braces.	Direct bonding of braces on teeth 1, 3, 4, and 6 and opening of self-ligating braces.	Direct bonding of braces on teeth 1, 3, 4, and 6 and opening of self-ligating braces.
	2	DA	Assisting: coat brackets with composite and apply, cure with UV lamp.	Assisting: coat brackets with composite and apply, cure with UV lamp.	Assisting: coat brackets with composite and apply, cure with UV lamp.	Assisting: coat brackets with composite and apply, cure with UV lamp.
	3	D	Insertion of the archwire.	Insertion of the archwire.	Insertion of the archwire.	Insertion of the archwire.
	3	DA	Pre-shortening of the archwire.	Pre-shortening of the archwire.	Pre-shortening of the archwire.	Pre-shortening of the archwire.
	4	D	Integration of bracket 3 using elastic ligation.	Integration of bracket 3 using elastic ligation.	Integration of bracket 3 using elastic ligation.	Integration of brackets 3 using elastic ligation.
	4	DA	Picking up and applying Alastic with needle holder.	Picking up and applying Alastic with needle holder.	Picking up and applying Alastic with needle holder.	Picking up and applying Alastic with needle holder.
	5	D	Integration of brackets 1 and 4 using metal ligation.	Integration of brackets 1 and 4 using metal ligation.	Integration of brackets 1 and 4 using metal ligation.	Integration of brackets 1 and 4 using metal ligation.
	5	DA	Assisting: picking up and applying metal ligature with needle holder.	Assisting: picking up and applying metal ligature with needle holder.	Assisting: picking up and applying metal ligature with needle holder.	Assisting: picking up and applying metal ligature with needle holder.
	6	D	Debonding of bracket.	Debonding of bracket.	Debonding of bracket.	Debonding of bracket.
	6	DA	Assisting: applying the tongs for debonding.	Assisting: applying the tongs for debonding.	Assisting: applying the tongs for debonding.	Assisting: applying the tongs for debonding.
Oral and maxillofacialsurgery			Surgical removal of tooth 13	Surgical removal of tooth 23	Surgical removal of tooth 38	Surgical removal of tooth 48
	1	D	Palatinal and marginal incision in regions 16 to 11.	Vestibular and marginal incision in regions 21 to 25.	Crestal incision in region 38 with a mesial relieving incision.	Crestal incision in regions 48 to 44.
	1	DA	Suctioning with the small suction cup.	Suctioning with the small suction cup.	Suctioning with the small suction cup.	Suctioning with the small suction cup.
	2	D	Exposure of the palatinal impacted tooth 13 by osteotomy using a surgical round bur. If necessary, cut through the tooth using a Lindemann bur. Removal of the tooth 13 using a Bein root elevator or dental forceps. Curettage of the dental sac.	Exposure of the vestibular impacted tooth 23 by osteotomy using a surgical round bur. If necessary, cut through the tooth using a Lindemann bur. Removal of the tooth 23 using a Bein root elevator or dental forceps. Curettage of the dental sac.	Exposure of the impacted tooth 38 by osteotomy using a surgical round bur. Removal of the tooth 38 using a Bein root elevator or dental forceps. Curettage of the dental sac.	Exposure of the impacted tooth 48 by osteotomy using a surgical round bur. If necessary, cutting through the tooth using a Lindemann bur. Removal of the tooth 48 using a Bein root elevator or dental forceps. Curettage of the dental sac.
	2	DA	Aspirating and holding of the flap with an instrument of choice, handing instruments as requested by the dentist.	Aspirating and holding of the flap with an instrument of choice, handing instruments as requested by the dentist.	Aspirating and holding of the flap with an instrument of choice, handing instruments as requested by the dentist.	Aspirating and holding of the flap with an instrument of choice, handing instruments as requested by the dentist.
	3	D	Wound closure with single loop interrupted sutures.	Wound closure with single loop interrupted sutures.	Wound closure with single loop interrupted sutures.	Wound closure with single loop interrupted sutures.
	3	DA	Clamping the needle in the needle holder and cutting the seam with the scissors.	Clamping the needle in the needle holder and cutting the seam with the scissors.	Clamping the needle in the needle holder and cutting the seam with the scissors.	Clamping the needle in the needle holder and cutting the seam with the scissors.

**Table 3:** RULA scores for dentists and dental assistants of oral and maxillofacial surgery, endodontology, general dentistry, and orthodontics. Significant differences between dental assistants and dentists are indicated in asterisks, whereby the significant lower ergonomic risk score is marked. In addition, asterisks indicate the magnitude of the statistical significance (* *p* < 0.05; ** *p* < 0.01; *** *p* < 0.001).

RULA Score	Occupation	Oral and Maxillofacial Surgery			Endodontology			General Dentistry			Orthodontics		
		Median (IQD)	Rel. Av. RST	ERP (%)	Median (IQD)	Rel. Av. RST	ERP (%)	Median (IQD)	Rel. Av. RST	ERP (%)	Median (IQD)	Rel. Av. RST	ERP (%)
Final right (Max. Score 7)	DA	5 (1)	5.19	74.12	4.25 (1)	4.66	66.54	5 (1)	5.23	74.70	5 (1)	4.75	67.91
	D	5 (1) *	5.00	71.49	5 (1)	4.87	69.51	5 (1)	5.05	72.13	5 (1)	4.66	66.57
Final left (Max. Score 7)	DA	5.5 (1)	5.22	74.59	4 (1)	4.63	66.11	5.5 (1)	5.26	75.15	5 (1)	4.81	68.78
	D	5 (1) *	4.98 *	71.13	5 (1)	4.78	68.35	5 (1)	5.19	74.10	4 (1)	4.49	64.08
Trunk Position-Step 10	DA	3 (1)	2.66	44.28	2 (0)	2.19	36.58	2 (0.75) *	2.30	38.25	2 (0)	2.17	36.21
	D	2.50 (1)	2.52	42.08	2.5 (1)	2.51	41.81	3 (0.75)	2.73	45.47	2 (0.75)	2.32	38.73
Neck Position-Step 9	DA	3.5 (0.5)	3.68	61.42	3 (1)	3.45	57.56	3.75 (1)	3.63	60.49	3.5 (1)	3.62	60.37
	D	3 (1)	3.48	57.98	3 (1)	3.46	57.63	3.25 (1)	3.52	58.69	3 (1) *	3.37 **	56.21
Right Wrist-Steps 3 and 4	DA	4 (0.75)	4.20	70.01	5 (1)	4.52	75.31	4.75 (1)	4.56	76.05	4 (0.75)	4.17	69.46
	D	4 (1)	4.34	72.3	4 (1) **	4.20 *	70.01	4.5 (1)	4.51	75.20	4 (1)	4.31	71.84
Right Lower Arm-Step 2	DA	2 (1)	2.45	81.73	2.25 (1)	2.45	81.73	2.25 (1)	2.37	79.07	3 (1)	2.69	89.71
	D	2(1)	2.34	78.13	2(1)	2.31	76.92	2 (0) *	2.12 **	70.77	2 (1) **	2.41 ***	80.35
Right Upper Arm-Step 1	DA	2 (0.75)	1.74 **	28.96	2 (1)	1.67 *	27.78	2 (0.5)	1.79	29.85	2 (1)	1.78	29.66
	D	2 (0.25)	1.89	31.55	2(1.25)	1.91	31.80	2 (0.5)	1.90	31.68	2 (1)	1.73	28.84
Left Wrist-Steps 3 and 4	DA	4.5 (0.5)	4.47	74.45	4 (1)	4.20	69.99	4.5 (1)	4.35	72.52	4 (1)	4.23	70.49
	D	5 (1)	4.53	75.52	4 (1)	4.33	72.23	5 (0.75)	4.71	78.53	4 (0.5)	4.17	69.49
Left Lower Arm-Step 2	DA	2 (1)	2.37	79.17	2.25 (1)	2.40	80.00	2.25 (1)	2.38	79.25	3 (1)	2.61	87.07
	D	3 (1)	2.66	88.77	2 (1)	2.42	80.73	2.5 (1)	2.40	79.94	2 (1) **	2.28 ***	76.01
Left Upper Arm-Step 1	DA	1.5 (0.5)	1.56	26.06	1.5 (1)	1.49	24.88	1.5 (1)	1.63	27.22	1.5(1)	1.59	26.43
	D	1.5 (1)	1.51	25.11	1.5 (1)	1.55	25.87	1 (0.75)	1.36	22.59	2 (1)	1.71	28.48

Rel. av. RST = relative average risk score over the time; ERP = ergonomic risk potential; DA = Dental Assistant; D = Dentist.

## Data Availability

There are no further data or materials than shown in this manuscript.
